# Editorial: New Challenges in Globalized Societies: Cross-Cultural Studies and Test Adaptation

**DOI:** 10.3389/fpsyg.2022.900535

**Published:** 2022-05-10

**Authors:** Fco. Pablo Holgado-Tello, Salvador Chacón-Moscoso, Susana Sanduvete-Chaves, José Antonio Lozano-Lozano

**Affiliations:** ^1^National University of Distance Education (UNED), Madrid, Spain; ^2^Universidad Autónoma de Chile, Santiago, Chile; ^3^Universidad de Sevilla, Seville, Spain

**Keywords:** psychometrics, cross-cultural studies, tests adaptation, validity, analytical strategies

Tests have become one of the basic tools to carry large-scale psychological and educational evaluations. In globalized societies, having common elements of evaluation allows the comparison and measurement of participants from diverse cultures and backgrounds, with assurances that the instruments are free of bias, and consequently, that the assessments are objective. However, obtaining such instruments involves overcoming serious drawbacks that arise in their development. Among other aspects, these disadvantages are mainly due to the cultural origin of the populations to be measured with the same instrument.

In practice, this question involves considering all aspects, from the connotative and denotative meaning of each word to the very interpretation of the test scores. That is why it is not enough to translate literally the items of an already validated test in one population and apply them directly in a different language and culture. Instead, in addition to guarantee certain linguistic equivalences or construct, as well as developing new standards, it is necessary to obtain empirical evidence of validity again, in the broad sense of the term, in the new population.

The relevance of this issue was revealed in 1985, when the American Educational Research Association (AERA), together with the American Psychological Association (APA) and the National Council on Measurement in Education (NCME) published the Standards for Educational and Psychological Testing. It provides a theoretical framework to assist in the process of test adaptation and, although today they have been modified and completed with the new guidelines developed by the International Test Commission (ITC), it served to warn the possible sources of error that may arise during the adaptation process (Barbero et al., 2008). The ITC regularly publishes and updates the standards for the adaptation of tests. In the latest 2017 version, we can find a checklist of the fundamental aspects to consider (Hernández et al., 2020). However, although the importance and relevance of the problem were made explicit more than 30 years ago, a study by Rios and Sireci (2014) shows that most publications that propose adaptations do not follow ITC standards.

In this Research Topic, we would like to address the importance of test adaptations, which could imply to investigate the psychometric properties on different samples without the need of items translation, or studies that translate the test to a different language with a different sociocultural background. The relevance of this problematic is so high that authors from four different continents (Asia, Europa, America and Africa) have submitted their manuscripts contributing to reach an interesting Research Topic formed by different applications and techniques implied in test construction and adaptation. Within the most commonly used techniques in the general research about test construction and adaptation, it is significant the Confirmatory Factor Analysis and those derived from it, such as multigroup analysis, exploratory structural equation modeling (ESEM), or bifactor models.

Within the adaptation process, and in line with the fast changes that characterize societies nowadays, new challenges are appearing for psychometricians. In this sense, Psychometrics is undergoing its own developments, and as Ruhe and Zumbo (2009) point out, they affect and revitalize the framework of their classical test theories. Measurement in general, and Psychometrics in particular, are significantly affected by the emergence of modern technologies and methodologies such as information technology and the Internet which, among other aspects, allow for example the presentation of multimedia items, tele-assessment, or experience sampling methodology (Myin-Germeys and Kuppens, 2022). These new contexts and assessment formats also imply, to a greater or lesser degree, certain adaptation process that guarantees that the interpretations of the scores are free of artifactual bias.

The concrete contributions to this Research Topic present, in the context of on-line evaluation, empirical validity evidence of a short version of an instrument designed to assess the precompetitive psychological state of the athletes (Diaz-Tendero et al.). Additionally, differences between amateur and professional athletes were found based on on-line assessment. As mentioned before, this kind of assessment is highly facilitated by the irruption of methodologies as Internet and smart phones, which are going to overcome some of the traditional criticisms about the assessment using tests. Assessing the athletes just before the competition increases notably the ecological validity.

Precisely in the assessment process, one of the challenges that psychometricians must face is how to measure and obtain data in daily life in the real world in real time. In this sense, Fuster-RuizdeApodaca et al., step by step, according to the guidelines for test construction, have developed an useful and relevant measurement instrument to be applied in routine specialist clinical care to identify, in real time and in the real world, people living with HIV that could be suffering health-related issues referred to stigma, emotional distress, sexuality, social support, material deprivation, sleep, cognitive problems or physical symptoms.

To generalize the use of specific instruments, Zhang and Bian confirmed the measurement invariance of the two-factor structure of a test to measure emotion regulation across male and female college students in China. Additionally, Pina et al. did not found differences in math performance across gender in Chile and Spain.

To obtain validity evidence of the use of tests in specific countries, Goessmann et al. created a scale to measure intimate partner violence for the specific casuistic found in women in Iraq. Salamon et al. obtained a factor structure in a scale to measure work engagement in Hungary, consisted in a global factor and three co-existing specific factors of vigor, dedication, and absorption. Samfira and Maricutoiu assessed the psychometric properties of an inventory to measure perfectionism in Romanian teachers. Additionally, Hill et al. obtained validity evidence of an inventory to measure personality in South Africa.

Finally, in the context of test adaptation across cultures, Lacko et al. obtained validity evidence in the Czech version of a questionnaire to measure individualism/collectivism. Klocek et al. studied the psychometric properties of the Czech version of a scale to measure group cohesiveness. Sahagún-Morales et al. adapted two tools to measure potential child abuse and protective factors to the Mexican population. Furthermore, Xiong et al. adapted a survey to measure safety cognition capability to the Chinese population.

The editors greatly appreciate the contributions received from the authors in this Research Topic. We hope it will be of interest to readers, and that potential researchers will find motivation for the development of future work.

## Author Contributions

FH-T, SC-M, SS-C, and J-LL contributed to documenting, designing, drafting, writing the manuscript, and revised it for important theoretical and intellectual content. All authors provided final approval of the version to be published and agree to be accountable for all aspects of the work in ensuring that questions related to the accuracy or integrity of any part of the work are appropriately investigated and resolved.

## Funding

This work was supported by the Fondo Nacional de Desarrollo Científico y Tecnológico FONDECYT Regular, ANID, Chilean Government (1190945), the ERDF government program for Andalusia 2014-2020, Spain (US-1263096), the VI Research and Transfer Plan-VI-PPITUS, Universidad de Sevilla, Spain (VIPP PRECOMPETI 2020/1333), and the PID2020-115486GB-I00 grant funded by MCIN/AEI/10.1309/501100011033.

## Conflict of Interest

The authors declare that the research was conducted in the absence of any commercial or financial relationships that could be construed as a potential conflict of interest.

## Publisher's Note

All claims expressed in this article are solely those of the authors and do not necessarily represent those of their affiliated organizations, or those of the publisher, the editors and the reviewers. Any product that may be evaluated in this article, or claim that may be made by its manufacturer, is not guaranteed or endorsed by the publisher.
